# Performance of an automated registration-based method for longitudinal lesion matching and comparison to inter-reader variability

**DOI:** 10.1088/1361-6560/acef8f

**Published:** 2023-08-28

**Authors:** Daniel T Huff, Victor Santoro-Fernandes, Song Chen, Meijie Chen, Carl Kashuk, Amy J Weisman, Robert Jeraj, Timothy G Perk

**Affiliations:** 1 AIQ Solutions, Madison, WI, United States of America; 2 University of Wisconsin-Madison, Department of Medical Physics, Madison, WI, United States of America; 3 The First Hospital of China Medical University, Department of Nuclear Medicine, Shenyang, Liaoning, CN, People’s Republic of China; 4 University of Ljubljana, Faculty of Mathematics and Physics, Ljubljana, SI, Slovenia

**Keywords:** PET/CT, longitudinal, inter-reader variability, reproducibility

## Abstract

*Objective.* Patients with metastatic disease are followed throughout treatment with medical imaging, and accurately assessing changes of individual lesions is critical to properly inform clinical decisions. The goal of this work was to assess the performance of an automated lesion-matching algorithm in comparison to inter-reader variability (IRV) of matching lesions between scans of metastatic cancer patients. *Approach.* Forty pairs of longitudinal PET/CT and CT scans were collected and organized into four cohorts: lung cancers, head and neck cancers, lymphomas, and advanced cancers. Cases were also divided by cancer burden: low-burden (<10 lesions), intermediate-burden (10–29), and high-burden (30+). Two nuclear medicine physicians conducted independent reviews of each scan-pair and manually matched lesions. Matching differences between readers were assessed to quantify the IRV of lesion matching. The two readers met to form a consensus, which was considered a gold standard and compared against the output of an automated lesion-matching algorithm. IRV and performance of the automated method were quantified using precision, recall, F1-score, and the number of differences. *Main results.* The performance of the automated method did not differ significantly from IRV for any metric in any cohort (*p* > 0.05, Wilcoxon paired test). In high-burden cases, the F1-score (median [range]) was 0.89 [0.63, 1.00] between the automated method and reader consensus and 0.93 [0.72, 1.00] between readers. In low-burden cases, F1-scores were 1.00 [0.40, 1.00] and 1.00 [0.40, 1.00], for the automated method and IRV, respectively. Automated matching was significantly more efficient than either reader (*p* < 0.001). In high-burden cases, median matching time for the readers was 60 and 30 min, respectively, while automated matching took a median of 3.9 min *Significance.* The automated lesion-matching algorithm was successful in performing lesion matching, meeting the benchmark of IRV. Automated lesion matching can significantly expedite and improve the consistency of longitudinal lesion-matching.

## 1. Introduction

Patients with metastatic cancers are often imaged longitudinally throughout the course of their disease for diagnosis, staging, and response assessment with a variety of modalities including computed tomography (CT), magnetic resonance imaging (MRI), or positron emission tomography (PET). Images are interpreted by clinicians to judge therapy efficacy and to make decisions about a patient’s treatment pathway. Central to the interpretation of longitudinal radiological images is the assessment of changes in lesions from one timepoint to the next. This includes the appearance of new lesions, the disappearance of lesions responding to treatment, and changes in the size or appearance of persistent lesions.

When treated systemically, metastatic cancers often exhibit lesion-wise heterogeneity in response, where some lesions disappear or shrink, some lesions remain stable, some lesions grow, and new lesions appear despite the ongoing treatment. This response pattern has been termed ‘dissociated response’, ‘mixed response’, and ‘heterogeneous response’, and has been observed in 21%–48% of solid cancers treated with chemotherapies and targeted therapies (Humbert and Chardin 2020). The response of individual lesions has been shown to drive progression. In particular, the appearance of new lesions can negatively impact patient outcomes (Harmon *et al* 2017). Thus, matching lesions between longitudinal images is critical to ensure that heterogeneous response patterns can be identified, and imaging data can be best utilized to inform treatment decisions.

Matching multiple lesions between longitudinal scans is a difficult task for clinicians to perform. Lesions may be numerous and densely packed within a single organ or tissue. For example, metastatic prostate cancer patients with high disease burden can exhibit over 100 lesions (Wang and Shen 2012). Additionally, lesions may grow, shrink, split, or merge over time. Patients may be imaged in different positions (e.g. with arms up or down, prone or supine, with knees bent or straight), and patient anatomy may change between images (e.g. weight loss due to treatment, surgical changes). Finally, patients may be imaged with different modalities (e.g. CT, PET/CT, MRI), they may be imaged on different scanners, and their images may be interpreted by different clinicians at each imaging timepoint. Inter-reader effects, such as differences in clinician experience, practice patterns, and reporting preferences, may result in inconsistencies in how changes in patient disease between imaging timepoints are captured and acted upon.

The result of these difficulties is that commonly used response criteria, such as the Response Evaluation Criteria in Solid Tumors (RECIST) (Eisenhauer *et al* 2009), consider only five target lesions to determine patient response. Matching of all lesions in a scan of a high-burden metastatic cancer patient is not performed as part of standard clinical practice, due to the amount of time and effort it would require, particularly when lesions respond heterogeneously. Without automated software tools, comprehensive lesion matching is not currently feasible for clinicians to perform for metastic cancer patients.

Inter-reader variability (IRV), also called inter-observer variability, is a well-established measure of reliability of medical image interpretation and analyses. A large portion of IRV studies in medical imaging have centred on image segmentation problems, such as delineation of prostate tumours on MRI (Steenbergen *et al* 2015), delineation of lung tumours on cone-beam CT (Sweeney *et al* 2012), and delineation of organs-at-risk for external beam radiation therapy of head and neck tumours on CT (Feng *et al* 2010). IRV has also been assessed in classification contexts including breast tumour feature analysis using the Breast Imaging Reporting and Data System (BIRADs) (Lee *et al* 2008) and target lesion identification and measurement according to the Response Evaluation Criteria in Solid Tumours (RECIST) (Muenzel *et al* 2012, Yoon *et al* 2016). Interventions designed to reduce IRV and increase consistency in image analysis and interpretation is an ongoing area of research (Vinod *et al* 2016, Tizhoosh *et al* 2021).

Inter-reader variability has been used as a benchmark for the evaluation of automated image analysis tasks. For example, the performance of an automated method for detecting lymphoma lesions on ^18^F-FDG PET/CT was benchmarked against the variability between two clinicians performing the same task (Weisman *et al* 2020). Turing tests, where users are asked to distinguish automated outputs from expert outputs, have been used to benchmark organ contouring performance (Gooding *et al* 2018). The rationale for using IRV as a performance benchmark is that IRV captures the variability plausibly present in any reference standard dataset against which the automated method is tested.

The objective of this study was to compare the performance of an automated lesion-matching method against the reference standard of IRV in the task of matching lesions between longitudinal PET/CT and CT scans. We hypothesized that the performance of the developed automated lesion-matching would be comparable to the IRV. The main contributions of this manuscript are: (1) the first head-to-head comparison of automated lesion matching with a reader-produced reference standard, and (2) the first reporting of IRV in the task of lesion matching.

## 2. Methods

### 2.1. Study population

Scan-pairs in four disease cohorts (lung, head and neck, lymphoma, and other advanced cancers) were collected for analysis. All data were collected either from public sources or obtained by AIQ Solutions, a biotechnology company that is developing a clinical decision support software for oncologists to manage late stage cancer patients, as part of research collaborations with academic medical centres. These cohorts were selected for their range of disease burden, and differences in spatial distributions of lesions. For some datasets, lesion contours were provided from the dataset source. For scans where contours were not provided, lesions were identified and segmented by author SC. All provided lesion contours were reviewed for accuracy by two nuclear medicine physician with 15 and 11 years’ experience (authors SC and MC) prior to completion of the lesion matching task. Lesions on each scan were assigned unique integer indices via connected component analysis.

#### 2.1.1. Non-small cell lung cancer

Ten subjects with metastatic non-small cell lung cancer (NSCLC) imaged with ^18^F-FDG PET/CT were randomly selected from a public dataset (ACRIN-NSCLC-FDG-PET: ACRIN 6668) (Kinahan *et al* 2019).

#### 2.1.2. Head and neck cancers

Ten subjects with head and neck cancers (squamous cell carcinomas) imaged with ^18^F-FDG PET/CT were randomly selected (*N* = 5 each) from two public datasets (QIN-HEADNECK (Beichel *et al* 2015), and HNSCC (Grossberg *et al* 2020)).

#### 2.1.3. Diffuse large B cell lymphoma

Ten subjects with diffuse large B-cell lymphoma (DLBCL) imaged with ^18^F-FDG PET/CT were randomly selected from a public dataset (CALGB-50503) (Bartlett *et al* 2020).

#### 2.1.4. Advanced cancers

Ten subjects with other advanced malignancies (metastatic neuroendocrine, prostate, breast, melanoma, and lung cancers) imaged with a variety of imaging modalities (PET/CT or CT) were collected for analysis. Patients were selected from a variety of internal and collaborator-provided sources specifically for having advanced disease to assess lesion matching IRV and performance in difficult cases.

Cases were also divided by the number of lesions into three disease-burden cohorts: low burden (<10 lesions), intermediate burden (10–29 lesions), and high burden (30+ lesions). The number of lesions was taken as the sum of the number of lesions on both scans.

Imaging data in the non-small cell lung cancer, heand and neck cancers, and diffuse large B cell lymphoma were all obtained from publiclly available datasets hosted by The Cancer Imaging Archive. Imaging data in the Advanced Cancers cohort were obtained from various AIQ collaborators, was anonymized prior to receipt by AIQ Solutions, and was shared with explicit permission for use in research projects. AIQ’s access to the retrospective imaging data followed all professional standards applicable to research including compliance for access to data including the protection of patient privacy.

### 2.2. Lesion matches as graphs

An undirected bipartite graph *G*(*N*_1_, *N*_2_, *E*) was used to describe lesion matches between a pair of scans, where nodes *N* represent lesions and edges *E* represent matches. For a scan pair, one group of nodes *N*_1_ = {*n*_1,1_, *n*_1,2_, …, *n*_1,*i*_} represents lesions on the first scan and a second group of nodes *N*_2_ = {*n*_2,1_, *n*_2,2_, …, *n*_2,*i*_} represents lesions on the second scan. Edges *E* between a node in the first scan and a node in the second scan represent a match. For example, if lesion 2 on the first scan matches to lesion 6 on the second scan, the edge *e* = {*n*_1,2_, *n*_2,6_} is added to the graph.

One extra node was added to each group to account for lesions which do not match (e.g. lesions which disappear or are new on the second scan). Lesions that disappear (present on the first scan but not the second) are accounted for with an edge *e* connecting the node for that lesion in the first scan to the added ‘disappeared’ node in the second scan *e* = {*n*_1,*i*_, *n*_2,disappeared_}. Lesions that are new on the second scan are accounted for with an edge connecting the node for that lesion in the second scan to the added ‘new’ node in the first scan e = {*n*_1,new_, *n*_2,*i*_}.

While our analyses in this study were limited to exactly 2 scans per subject, the lesion graph is generalizable to any number of scans. A series of *k* scans can be represented by a *k-*partite graph.

### 2.3. Automated lesion matching algorithm

An automated, registration-based lesion matching method was developed by our research group and has been reported in a previous publication (Santoro-Fernandes *et al* 2021). Briefly, the method consists of four steps: (1) image registration using 3D deformable registration of CT images (Rueckert 1999), (2) lesion dilation to account for registration uncertainties, (3) lesion clustering to account for lesions merging or splitting between scans, and (4) lesion matching via the Munkres assignment algorithm (Munkres 1957), which maximizes lesion intersection volume between scans.

The registration step (1) reported in Santoro-Fernandes *et al* (2021) has undergone additional refinement since the publication of Santoro-Fernandes *et al* (2021). First, bones and organs are contoured on the CT images using a previously trained convolutional neural network (Weisman *et al* 2022a, 2022b). Next, initial alignment of the two scans is performed via a rigid (translation only) registration of the organ and bone masks. Finally, a deformable registration is performed using a free-form deformation based on B-splines. All registration was performed using SimpleElastix software (Marstal *et al* 2016). The dilation step (2) utilized a fixed dilation magnitude of 25 mm, as was determined to be optimal for lesion matching in our previous study (Santoro-Fernandes *et al* 2021).

The automated lesion matching method was used to match lesions for all scan pairs. Matches produced by the automated method were compared against the reader consensus. Automated lesion matching was run twice for each scan pair to evaluate the reproducibility of automated lesion matching. The amount of time taken by the automated method was also recorded. Automated lesion matching was performed on a desktop workstation with an 8 core/16 thread CPU and 16 GB of RAM.

### 2.4. Multi-reader lesion matching study

Two nuclear medicine physicians with 15 and 11 years experience (authors SC and MC) performed the lesion matching task. For each scan pair, each reader was provided with images (PET/CT or CT) and lesion contours where each lesion was labeled with a unique integer index. Matching review was completed using 3D Slicer, an open-source platform for medical image viewing and analysis (Kikinis *et al* 2014). Readers were also provided with a spreadsheet workbook to record their matching results. For each scan pair readers filled two columns, where the first column listed lesion indices present in the first scan, and the second column listed lesion indices present in the second scan. Each row thus described lesion correspondence between the two scans. Lesions matched between scans were recorded by putting the corresponding lesion indices in both columns. Lesions present in only one scan (not matched) were noted by a zero (0) in the column corresponding to the scan on which the lesion was not present. Readers also recorded the amount of time they took to review and match each scan-pair.

Following independent review of all cases, the two readers met to discuss all cases and reach a single expert consensus. The expert consensus was used as a reference standard against which the performance of the automated lesion matching method was compared.

### 2.5. Metrics for assessing lesion-matching algorithm performance and IRV

Inter-reader variability was assessed by comparing matches produced by reader A against matches produced independently by reader B. The matches of each reader were described as a graph *G*(*N*_1_, *N*_2_, *E*). Each reader produced one graph per subject. Graphs from two readers have identical nodes *N*_1_ and *N*_2_, but different sets of edges (*E*_*A*_ versus *E*_*B*_). IRV was thus assessed by comparing the set of edges *E*_*A*_ from the lesion matching graph produced by reader A *G*_*A*_(*N*_1_, *N*_2_, *E*_*A*_) against the set of edges *E*_*B*_ from the lesion graph produced by reader B *G*_*B*_(*N*_1_, *N*_2_, *E*_*B*_). Matching analyses were limited to lesions above a volume threshold of 0.1 cm^3^. This cutoff was chosen following discussion with the study readers (authors SC and MC). Readers were not confident in the reliability of lesion contours, or in their ability to reliably match lesions below a volume of 0.1 cm^3^.

Performance of the automated lesion matching method was assessed similarly, comparing the set of edges in the graph produced by the automated method *G*_auto_(*N*_1_, *N*_2_, *E*_auto_) against the set of edges in the graph produced by the reader consensus *G*_cons_(*N*_1_, *N*_2_, *E*_cons_). Both IRV and automated matching performance were quantified using the metrics outlined as follows.

**Precision**—the proportion of matches present in reader *A*’s matches that were also present in reader *B*’s matches. This is also called positive predictive value (PPV):

\begin{eqnarray*}P\left({E}_{A},{E}_{B}\right)=\frac{\left|{E}_{A}\cap {E}_{B}\right|}{\left|{E}_{A}\right|}.\end{eqnarray*}

**Recall**—the proportion of matches present in reader *B*’s matches that were also present in reader *A*’s matches. Also called sensitivity:

\begin{eqnarray*}R\left({E}_{A},{E}_{B}\right)=\frac{\left|{E}_{A}\cap {E}_{B}\right|}{\left|{E}_{B}\right|}.\end{eqnarray*}

**F1 score**—the harmonic mean of precision and recall:

\begin{eqnarray*}F\left({E}_{A},{E}_{B}\right)=2\frac{P\left({E}_{A},{E}_{B}\right)R({E}_{A},{E}_{B})}{P\left({E}_{A},{E}_{B}\right)+R({E}_{A},{E}_{B})}=\frac{2\left|{E}_{A}\cap {E}_{B}\right|}{2\left|{E}_{A}\cap {E}_{B}\right|+\left|{E}_{A}/{E}_{B}\right|+\left|{E}_{B}/{E}_{A}\right|}.\end{eqnarray*}

**Number of differences** *N*_d_—the number of edges present in one graph and not the other. This is equivalent to the cardinality of the symmetric difference between the sets of edges *E*_*A*_ and *E*_*B*_

\begin{eqnarray*}{N}_{d}({E}_{A},{E}_{B})=\left|{E}_{A}/{E}_{B}\right|+\left|{E}_{B}/{E}_{A}\right|.\end{eqnarray*}

For assessing the performance of the automated lesion matching method versus the reference standard reader consensus, we set *E*_*A*_ = *E*_auto_ and *E*_*B*_ = *E*_cons_, where *E*_auto_ and *E*_cons_ were the sets of edges produced by the automated matching method and the reader consensus, respectively. For the assessment of IRV, we adopted the convention for precision and recall that reader B’s matches were the reference standard against which reader A’s matches were being evaluated. This choice was arbitrary, and if it were to be reversed, the effect would be that the reported values for IRV precision and recall would be reversed. The F1 score and the number of differences *N*_d_ would be equivalent if the order of readers A and B were reversed (e.g. *F*(*E_A_*, *E_B_*) = *F*(*E_B_*, *E_A_*)).

### 2.6. Statistical analysis

Differences between IRV and performance of the automated method and differences in matching time were assessed with paired Wilcoxon tests. Correlation between lesion matching metrics, the time for readers to perform matching, and the number of lesions in each scan-pair were assessed with Spearman correlation.

## 3. Results

### 3.1. Automated lesion matching

Clinical characteristics of the dataset are reported in table 1. Automated matching performance by disease-cohort is shown in table 2. Automated lesion matching performance was not significantly different from IRV for any assessed metric, for any disease-cohort (Wilcoxon paired test, *p* > 0.05). However, when all *N* = 40 cases were considered, a significant difference in Recall between IRV and automated matching performance was observed (IRV: median recall of 1.00, automated: median recall of 0.92, *p* = 0.05). A similar difference in the number of differences was verging on significance at the *α* = 0.05 level (IRV: median *N*_d_ of 0, automated: median *N*_d_ of 2, *p* = 0.06). In the Advanced Cancers disease cohort (41.6 ± 43.0 lesions per scan), at least one difference in matching between the automated method and reader consensus was observed in 8/10 (80%) of cases.

**Table 1.** Patient characteristics. NSCLC = non-small cell lung cancer.

	NSCLC (*N* = 10)	Head and neck (*N* = 10)	Lymphoma (*N* = 10)	Advanced cancers (*N* = 10)
Sex—*n* (%)				
Male	6 (60%)	6 (60%)	6 (60%)	8 (80%)
Female	1 (10%)	4 (40%)	4 (40%)	2 (20%)
Not provided	3 (30%)	0 (0%)	0 (0%)	0 (0%)
Age—year				
Median (range)	61 (47, 69)	58 (48, 66)	51 (36, 74)	67 (48, 77)
Disease stage				
1	0	0	1	0
2	1	2	1	0
3	6	2	1	2
4	0	6	7	8
Not provided	3	0	0	0
Treatment	Platinum-based chemoradiotherapy without surgery	Chemoradiotherapies, various	Rituximab plus chemotherapies	Various (Lu-radiopharmaceutical therapies, hormonal therapy, immunotherapies, chemotherapies)
Time between scans—days				
Median (range)	162 (82, 210)	172.5 (102, 312)	115 (44, 156)	95 (0, 912)

**Table 2.** Inter-reader variability of lesion matching versus the performance of the automated lesion matching method (auto) by disease cohorts. Data are reported as median (range). *P*-values are tests for significant differences between IRV and automated matching performance (Wilcoxon paired tests).

	Precision	Recall	F1 score	*N*_d_
NSCLC (*N* = 10)				
IRV	0.97 (0.67, 1.00)	1.00 (0.50, 1.00)	0.98 (0.57, 1.00)	0.5 (0, 4)
Auto	0.92 (0.80, 1.00)	0.89 (0.71, 1.00)	0.91 (0.75, 1.00)	2.5 (0, 8)
*p*	0.74	0.26	0.40	0.18
Head and neck (*N* = 10)				
IRV	1.00 (1.00, 1.00)	1.00 (1.00, 1.00)	1.00 (1.00, 1.00)	0 (0, 0)
Auto	1.00 (0.50, 1.00)	1.00 (0.33, 1.00)	1.00 (0.40, 1.00)	0 (0, 3)
*p*	0.11	0.11	0.11	0.08
Lymphoma (*N* = 10)				
IRV	1.00 (0.25, 1.00)	1.00 (1.00, 1.00)	1.00 (0.40, 1.00)	0 (0, 3)
Auto	1.00 (0.92, 1.00)	1.00 (0.80, 1.00)	1.00 (0.86, 1.00)	0 (0, 4)
*p*	1.00	0.18	1.00	0.41
Advanced Cancers (*N* = 10)				
IRV	0.92 (0.69, 1.00)	0.86 (0.74, 0.96)	0.89 (0.72, 0.96)	5.5 (2, 58)
Auto	0.88 (0.59, 1.00)	0.86 (0.60, 1.00)	0.87 (0.63, 1.00)	15.5 (0, 59)
*p*	0.24	0.95	0.86	0.53
ALL (*N* = 40)				
IRV	1.00 (0.25, 1.00)	1.00 (0.50, 1.00)	1.00 (0.40, 1.00)	0 (0, 58)
Auto	0.97 (0.50, 1.00)	0.92 (0.33, 1.00)	0.94 (0.40, 1.00)	2 (0, 59)
*p*	0.14	0.05	0.12	0.06

The performance of the automated lesion matching method was dependent on disease burden. In high-burden cases (30+ lesions, *N* = 9 cases), median F1-score was 0.89, and one or more differences in matching was observed in 8/9 (89%) cases. In low-burden cases (<10 lesions, *N* = 14), the median F1-score was 1.00, and one or more differences in matching was observed in 2/14 (14%) cases. Performance of the automated matching method by disease burden is summarized in table 3. Automated lesion matching performance was not significantly different from IRV for any assessed metric, for any burden-cohort (Wilcoxon paired test, *p* > 0.05).

**Table 3.** Inter-reader variability of lesion matching versus the performance of the automated lesion matching method (auto) by disease burden. Cases were divided into three disease-burden cohorts: low-(<10 lesions), intermediate- (10–29 lesions) and high- (30 or more lesions) burden. Data are reported as median (range). *P*-values are tests for significant differences between IRV and automated matching performance (Wilcoxon paired tests).

	Precision	Recall	F1 score	*N*_d_
Low burden (*N* = 14)				
IRV	1.00 (0.25, 1.00)	1.00 (0.50, 1.00)	1.00 (0.40, 1.00)	0 (0, 3)
Auto	1.00 (0.50, 1.00)	1.00 (0.33, 1.00)	1.00 (0.40, 1.00)	0 (0, 3)
*p*	0.85	0.41	1.00	1.00
Intermediate burden (*N* = 17)				
IRV	1.00 (0.80, 1.00)	1.00 (0.77, 1.00)	1.00 (0.79, 1.00)	0 (0, 7)
Auto	0.92 (0.59, 1.00)	0.91 (0.71, 1.00)	0.91 (0.65, 1.00)	2 (0, 14)
*p*	0.17	0.27	0.17	0.11
High burden (*N* = 9)				
IRV	0.95 (0.69, 1.00)	0.91 (0.74, 1.00)	0.93 (0.72, 1.00)	5 (0, 58)
Auto	0.91 (0.66, 1.00)	0.86 (0.60, 1.00)	0.89 (0.63, 1.00)	17 (0, 59)
*p*	0.12	0.26	0.26	0.18

**Figure 1.**
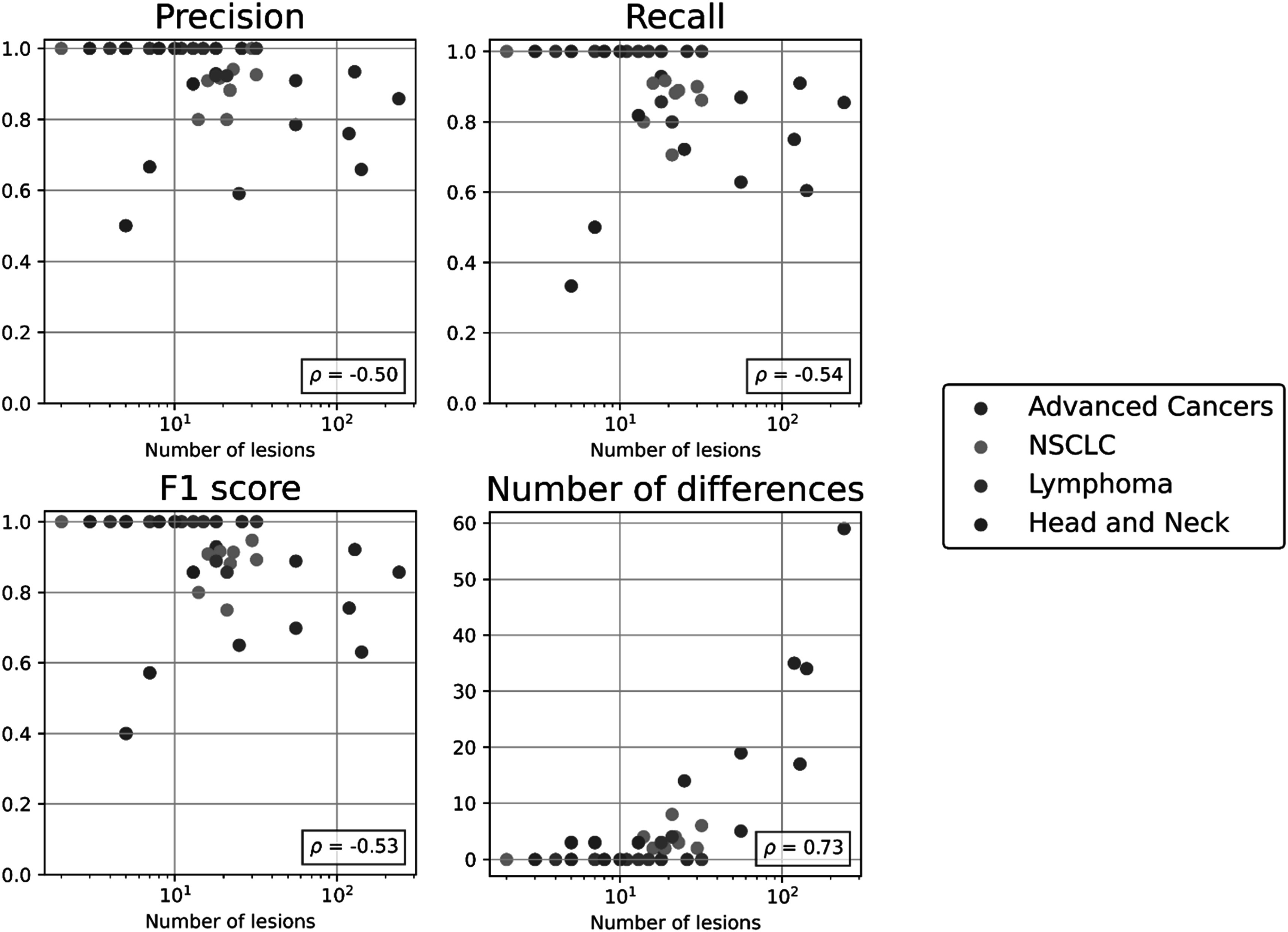
Performance of the automated lesion-matching algorithm as a function of the number of lesions in four cohorts. Number of lesions is defined as the sum of the number of lesions present on the two scans in each case. Each metric is annotated with Spearman correlation (*ρ*) between the metric and the number of lesions. Note the data are presented on a log scale.

We investigated correlation between automated lesion matching metrics and the number of lesions per scan-pair. As the number of lesions increased, the performance of the automated matching decreased for all metrics (Spearman correlation, *p* < 0.05). Automated lesion matching metrics as a function of number of lesions are shown in figure 1.

### 3.2. Multi-reader lesion matching study

**Figure 2.**
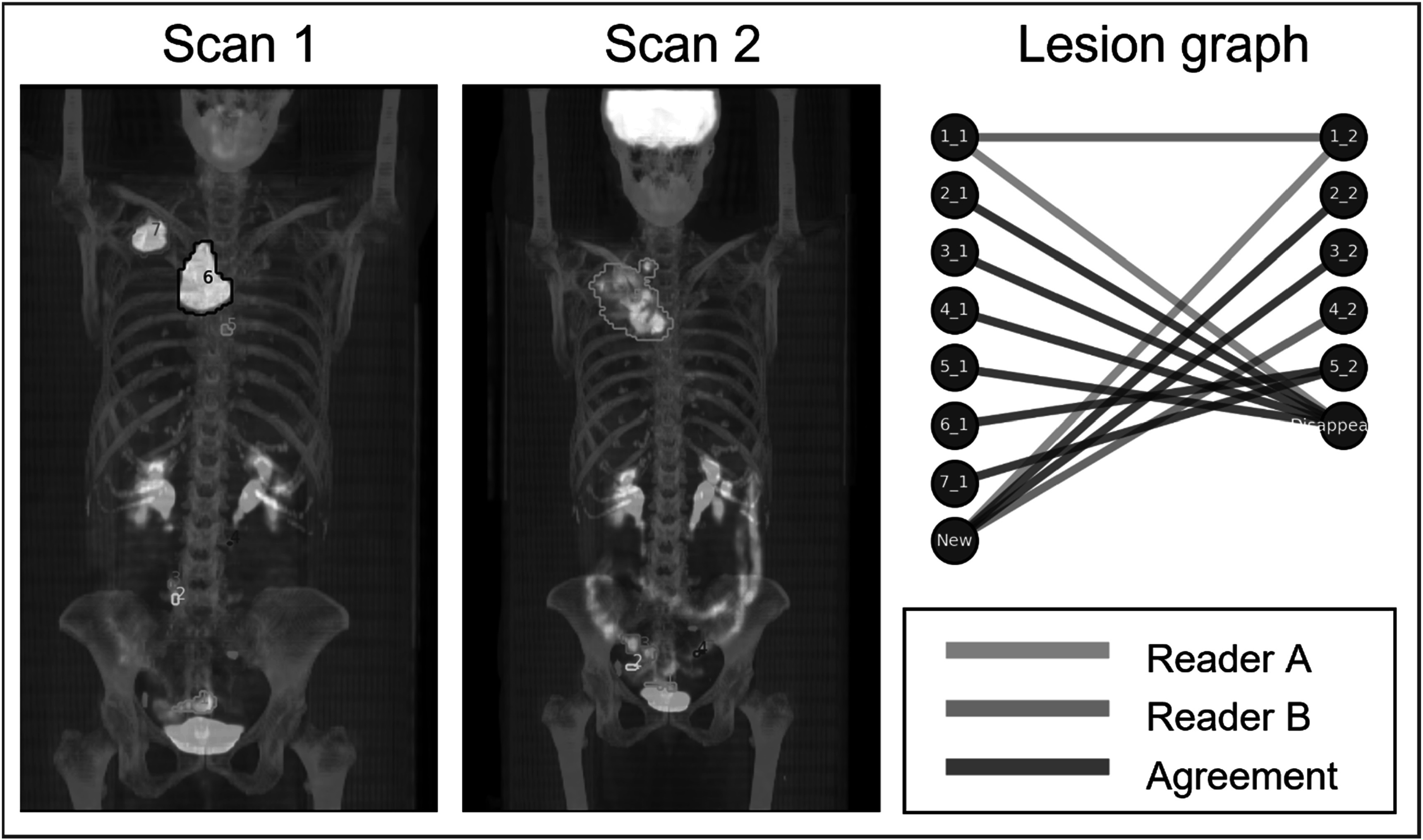
Lesion matching in a female subject with stage III NSCLC imaged with ^18^F-FDG PET/CT before (Scan 1) and after (Scan 2) platinum-based chemotherapy. PET/CT were acquired 179 d apart. The lesion located in the rectum (orange contour, label 1) is matched differently between readers. Reader A determines that the lesion disappears after scan 1 and a new lesion in a similar area appears on scan 2. Reader B determines that these two lesions are homologous and should be matched between scans.

Both readers completed independent review of all *N* = 40 scan-pairs and recorded matching results. An example of the inter-reader lesion matching analysis for a subject in the NSCLC cohort is shown in figure 2. A full summary of inter-reader variaibility by disease-cohort is shown in table 2. In the Advanced Cancers disease cohort, at least one difference in matching between readers was observed in 10/10 (100%) cases.

Similar to the automated method, IRV was highly dependent on disease burden. In high-burden cases (30+ lesions, *N* = 9 cases), the median F1-score between the two readers was 0.93. One or more differences in matching was observed in 6/9 (67%) cases. In low-burden cases (<10 lesions, *N* = 14), the median F1-score between the two readers was 1.00. One or more differences in matching was observed in 2/14 (14%) of low-burden cases. IRV of lesion matching by disease burden is summarized in table 3.

**Figure 3.**
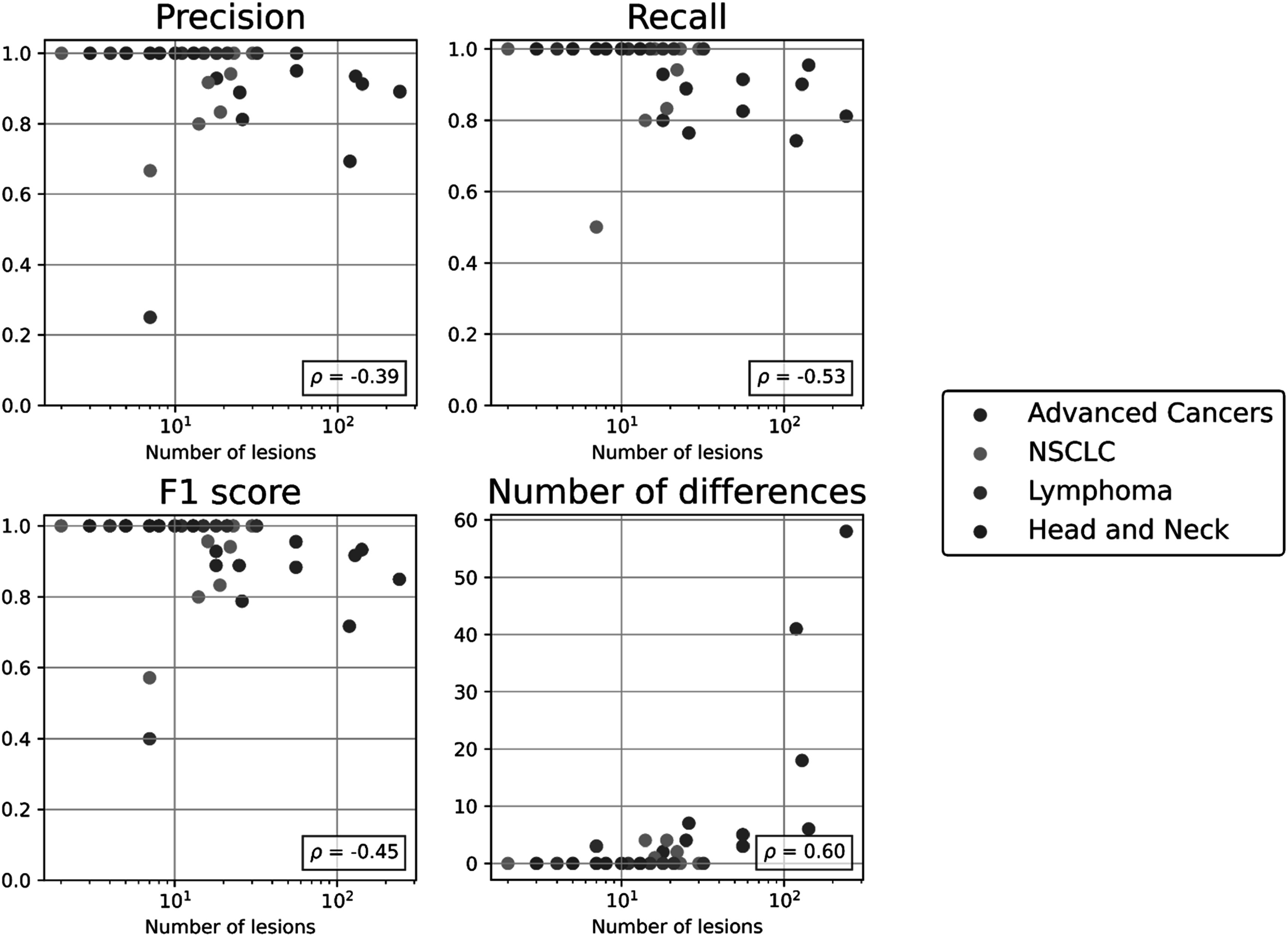
Matching IRV metrics as a function of the number of lesions in four cohorts. Number of lesions is defined as the sum of the number of lesions present on the two scans in each case. Each metric is annotated with Spearman correlation (*ρ*) between the metric and the number of lesions. Note the data are presented on a log scale.

We assessed correlation between IRV metrics and the number of lesions on each scan-pair (figure 3). Similar to automated matching performance, the amount of variation between readers increased as the number of lesions increased (Spearman correlation, *p* < 0.05) for all metrics.

### 3.3. Time spent on lesion matching

Across all *N* = 40 cases, manual, individual lesion matching by the two readers took a median of 5 (range: 1, 130) and 7 (range: 2, 120) minutes, respectively. The automated method took a median of 1.1 (range: 0.5, 10.6) minutes to match lesions. The automated lesion matching method took significantly less time to match lesions than either reader (Wilcoxon paired test, *p* < 0.001). The difference in matching time between readers was not significant (*p* = 0.37). Time to perform matching for the readers and automated method is summarized in table 4.

**Table 4.** Disease burden and time for readers and the automated method (Auto) to perform manual matching. Data are reported as median (range).

	Number of lesions—Scan 1	Number of lesions—Scan 2	Time reader A (min)	Time reader B (min)	Time auto (min)
NSCLC (*N* = 10)	8.5 (1, 28)	8.5 (1, 19)	10 (1, 20)	11.5 (4, 25)	1.1 (0.7, 1.7)
Head and neck (*N* = 10)	3 (2, 6)	3 (1, 7)	3 (2, 5)	3 (2, 6)	0.9 (0.5, 1.5)
Lymphoma (*N* = 10)	8 (2, 30)	3 (1, 8)	4 (2, 5)	4 (3, 16)	1.0 (0.8, 1.6)
Advanced cancers (*N* = 10)	21.5 (3, 63)	33.5 (7, 179)	60 (15, 130)	25 (10, 120)	3.9 (1.0, 10.6)
ALL (*N* = 40)	7 (1, 63)	6 (1, 179)	5 (1, 130)	7 (2, 120)	1.1 (0.5, 10.6)

In high-burden cases (30+ lesions, *N* = 9 cases), the median time to perform matching for the two readers was 60 and 30 min The automated method performed matching in high-burden cases in a median of 3.9 min In low-burden cases (<10 lesions, *N* = 14 cases), the median time to perform matching for the two readers was 3 and 3.5 min, and the corresponding time for the automated method was 0.9 min.

**Figure 4.**
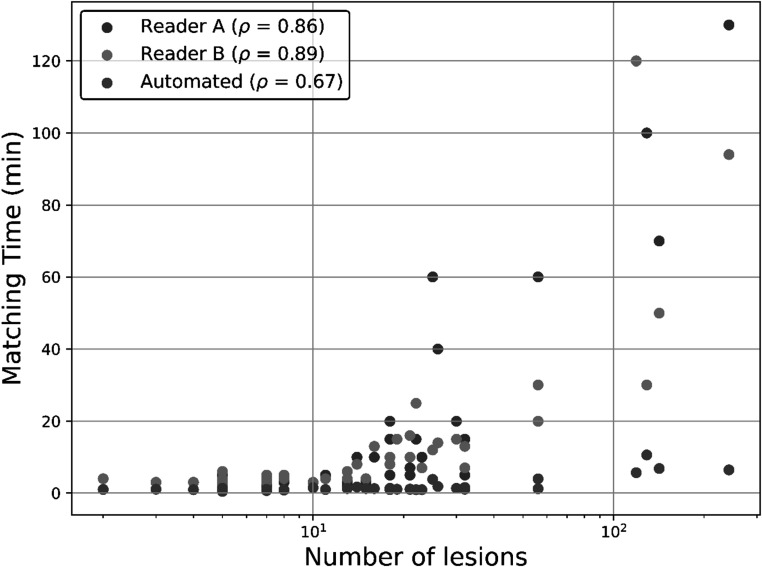
Time to perform manual matching for the two readers and the automated method as a function of the number of lesions in each matching case. Number of lesions is defined as the sum of the number of lesions present on the two scans in each case. Correlation between number of lesions and matching time was quantified with Spearman’s r. For all cases, correlation between number of lesions and matching time was significant (*p* < 0.001).

Positive correlation between the number of lesions in a scan pair and matching time for both readers (Spearman *ρ* = 0.86, *ρ* = 0.89), and for the automated lesion matching method (*ρ* = 0.67). Matching time as a function of number of lesions is shown in figure 4.

### 3.4. Reproducibility of automated lesion matching

To evaluate the reproducibility of the automated lesion matching algorithm, matching was performed twice for each scan pair and the matching results from the first run were compared to the results of the second run. No differences in lesion matching were observed between runs of the automated lesion matching algorithm (precision, recall, F1 score all =1 and *N*_d_ = 0).

## 4. Discussion

In this study, we assessed performance of an automated approach to lesion matching between longitudinal scans of patients with various metastatic cancers, and compared the performance of the automated method against IRV. When comparing the automated lesion matching method to the reader consensus as a reference standard, the automated lesion matching method performed within IRV. The performance of the automated lesion matching method was not significantly different from IRV of lesion matching for any assessed metric in any cohort.

Little IRV of lesion matching was observed in low burden cases (<10 lesions per scan). However, in high-burden cases (30+ lesions), differences between readers were seen in 67% of cases. In the advanced cancers disease cohort (up to 179 lesions per scan) selected specifically for matching difficulty, differences between readers were seen in 100% of cases. This suggests that IRV is of significant concern in patients with high disease burden imaged longitudinally. Moreover, this study represents only a single step of the image analysis that is performed for patients with cancer imaged longitudinally. Higher IRV would be observed if all steps in the analysis were included (i.e. lesion detection, segmentation, and response interpretation).

Across all cases, manual lesion matching took the two readers a median of 5 and 7 min, respectively. This was significantly longer than automated matching, which took a median of 1.1 min In high-burden cases, the difference between reader and automated lesion matching speed was most evident. Here, the readers took a median of 60 and 30 min, respectively, while the automated matching took a median of 3.9 min In current clinical practice, only a susbet of lesions may be matched between scans to perform a RECIST-based response assessment (Eisenhauer *et al* 2009). The high amount of time (up to 130 min) required for the readers in this study to perform lesion matching highlights why it is not performed in typical clinical practice today. Availablity of automated methods such as the one described in this study is important to enable access for clinicians to comprehensive lesion-matching in clinical practice with accurate, more reproducable results.

The readers who participated in the inter-reader study (authors SC and MC) have 15 and 11 years’ relevant experience and have contributed to the refinement of the automated lesion matching algorithm. They were also provided with precontoured and numbered lesion labels to perform matching. Due to their specific experience and provided lesion labels, they may perform lesion matching faster or more consistently than a typical clinician with less experience and who is not provided with precontoured lesion labels. This suggests that the estimate of time cost in our study may underestimate the true time cost of manual lesion matching if it were to be performed clinically.

For both readers and the automated method, significant correlation was observed between the number of lesions in the scans and the time to perform matching. While the difference in matching time between readers and the automated method was smaller for low-burden cases, automated lesion matching still conveys the inherent advantage of requiring zero reader time, excepting quality assurance. The speed of the automated method is dependent on the hardware of the computer it is executed on. In this study, we report the timing of the automated method running on a desktop workstation with an 8 core/16 thread CPU and 16 GB of RAM, which are reasonable specifications for a desktop workstation at the time of writing. Further speed improvements could be realized either through optimization of the automated code, or by executing the program on hardware with improved specifications.

When the automated lesion matching algorithm was run multiple times, no differences in matching results between runs were observed. This suggests the automated lesion matching algorithm is highly reproducible. Small differences in deformable image registration can occur between repeated trials, however these were minimized by using fixed random seeds, and were not substantial enough to result in differences in matching in our study. Therefore, the advantage of automated lesion matching is not only workflow time-saving, but also high reproducibility. The strength of matching reproducibilty may be especially relevant when matching is performed by less experienced operators. To validate this hypothesis, a multi-reader study using pairs of operators with a variety of experience levels could be performed.

We reviewed all cases where the performance of the automated lesion matching method deviated from the reader consensus matching. The most common reasons for deviations were: inaccurate image registration placing homologous lesions too far apart for a match to be established, small lesion fragments not being grouped with a nearby lesion cluster, and spurious matches being assigned between lesions which overlap following registration but occupy distinct tissues. These boundary conditions are of interest for future refinement of the automated lesion matching method. Based on these observations, it is likely that deformable image registration performance is the main factor affecting matching performance. Investigation of factors contributing to patient-specific registration undertainty, or alternative approaches to registration, such as deep learning-based registration (Fu *et al* 2020) should be performed. Beyond registration, further refinement of the method’s dilation step could be investigated by implementing anatomy-specific dilation magnitudes, as registration uncertainty in rigid anatomy such as bone is likely lower than uncertainty in soft tissue.

Readers took a maximum of 120 and 130 min to perform manual lesion matching. The highest average matching time occurred in a subject with metastatic neuroendocrine tumours, where each reader took 120 min to perform matching, and the automated method took 5.7 min This subject had 59 lesions on scan 1 and 60 lesions on scan 2, which were densely concentrated within the liver. This case was difficult for both the readers and the automated lesion matching method, resulting in an F1 score of 0.72 between readers and an F1 score of 0.76 between the automated method and reader consensus. Interestingly, while this case took the most reader time, it was not the case with the most lesions. The case with the most lesions was a subject with bone-metastatic prostate cancer imaged with CT, with 63 lesions on scan 1 and 179 lesions on scan 2. Readers took 130 and 94 min, respectively, to match this case, while the automated method took 6.5 min.

In this study, we analyzed lesion matches above a volume threshold of 0.1 cm^3^. This volume threshold was chosen in discussion with the two study readers, who were not confident in the reliability of lesion contours, or in their ability to reliably match lesions below a volume of 0.1 cm^3^. While such small lesions may represent only a small fraction of a patient’s overall disease burden, commonly used response criteria such as RECIST 1.1 define a response of Progressive Disease if any new lesions are noted, regardless of size (Eisenhauer *et al* 2009). For this reason, small lesions can impact patient management, and determining whether they are new or match to an existing lesion is of clinical consequence.

In our study, we used graph structures to describe the lesion matching problem and assess IRV of lesion matching. Several other published studies have made use of graphs to describe the process of following lesions over time. In Szeskin *et al* (2023), the authors use graphs to describe lesion matching, and report precision and recall of their dilation-based lesion matching approach of (mean ± sd) 0.86 ± 0.18 and 0.90 ± 015, respectively, which are similar to our results. Their dataset consisted of 50 scan-pairs containing a total of 492 lesions (mean of 9.8 lesions/subject), which is similar to our low-burden cohort. Their analysis was limited to liver lesions, and the did not assess IRV of lesion matching. In Yan *et al* (2018), a distance-based approach to lesion matching is evaluated in 103 patients imaged with CT. They report area under the precision–recall curve of 0.959, with an estimated precision and recall of 0.86 and 0.92, respectively. Their dataset contained 1313 lesions (mean of 12.7 lesions/subject), which is most similar to the intermediate-burden cohort in our study. Finally, ‘tumor trees’, which are graph structures, were used in Kuckertz *et al* (2022) to describe progression of tumor burden in longitudinally imaged cancer patients. The investigators use a spatial overlap criterion to determine matches, but do not evaluate the accuracy of their method.

Commonly used imaging response criteria such as the Response Evaluation Criteria in Sold Tumours (RECIST) assign patient response based on changes in a subset of visible lesions (Eisenhauer *et al* 2009). RECISTv1.1 assesses up to 5 target lesions to assign a response category. In our study, 14/40 cases (35%) of subjects had five or fewer lesions on both scans. Within this subset, 2/14 (14%) of cases contained one or more matching differences.

In this study, we assessed lesion matching in a population (*N* = 40) of patients with NSCLC, head and neck tumours, DLBCL, and various advanced cancers. These cohorts were selected for their range of disease burden, and differences in spatial distribution of lesions. Additionally, the data were collected retrospectively, and were not part of a prospective trial with the express purpose of conducting an inter-reader matching study.

## 5. Conclusion

The automated lesion-matching method met the benchmark of IRV, while performing the matching task significantly more efficiently than human readers. In low-burden patients, little to no IRV was observed and time cost for readers to perform lesion matching was acceptable. However, in higher-burden patients, substantial IRV was observed and time cost became incompatible with clinical workflow, highlighting the clinical utility of automated lesion matching.

## Data Availability

The data cannot be made publicly available upon publication because they are owned by a third party and the terms of use prevent public distribution. The data that support the findings of this study are available upon reasonable request from the authors.

## Funding

Research reported in this publication was partially supported by the National Cancer Institute of the National Institutes of Health under Award Number 2R44CA257253-02. The content is solely the responsibility of the authors and does not necessarily represent the official views of the National Institutes of Health.

## Ethical statement

Research was conducted in accordance with the principles embodied in the Declaration of Helsinki and in accordance with local statutory requirements. AIQ’s access to the retrospective imaging data followed all professional standards applicable to research including compliance for access to data including the protection of patient privacy.
